# Social Bonds and Exercise: Evidence for a Reciprocal Relationship

**DOI:** 10.1371/journal.pone.0136705

**Published:** 2015-08-28

**Authors:** Arran Davis, Jacob Taylor, Emma Cohen

**Affiliations:** 1 Institute of Cognitive and Evolutionary Anthropology, University of Oxford, 64 Banbury Road, Oxford, OX2 6PN, United Kingdom; 2 Wadham College, Parks Road, Oxford, OX1 3PN, United Kingdom; University of Exeter, UNITED KINGDOM

## Abstract

In two experimental studies, we investigated mechanisms hypothesized to underpin two pervasive and interrelated phenomena: that certain forms of group movement and exercise lead to social bonding and that social bonding can lead to enhanced exercise performance. In Study 1, we manipulated synchrony and exercise intensity among rowers and found that, compared with low intensity exercise, moderate intensity exercise led to significantly higher levels of cooperation in an economic game; no effect of synchrony vs. non-synchrony was found. In Study 2, we investigated the effects of bonding on performance, using synchrony as a cue of existing supportive social bonds among participants. An elite, highly bonded team of rugby players participated in solo, synchronized, and non-synchronized warm-up sessions; participants' anaerobic performance significantly improved after the brief synchronous warm-up relative to the non-synchronous warm-up. The findings substantiate claims concerning the reciprocal links between group exercise and social bonding, and may help to explain the ubiquity of collective physical activity across cultural domains as varied as play, ritual, sport, and dance.

## Introduction

From night clubbers and park runners to Sufi dervishes and Olympic athletes, people everywhere come together to move together. Physical activity, exercise, and sport have well-known positive effects on physical and psychological health. Social scientists have also long speculated about the benefits of such energetically costly activities for social cohesion [1,2], and a large body of research in social and sport psychology indicates a positive association between group cohesion and performance [3]. This suggests there may be a reciprocal relationship between group physical activity and social bonding.

Although group exercise (broadly construed) takes many forms in culture, it commonly involves coordination between co-actors and is performed at levels of intensity that are neither very low nor very high. There is increasing evidence that both level of intensity and behavioral coordination influence social bonding and performance. Sustained aerobic exercise at a moderate intensity (~70–85% of maximum heart rate)–but not low (~45%) or high (~90%) intensities—induces activity in the endocannabinoid (eCB) system [4] and similar results have been obtained in studies on the endogenous opioid system [5]. It has been proposed that the foraging success of human ancestors in persistence hunting and scavenging would have depended on distance-running abilities at a moderate, rather than low or high, intensity [4,6]. The observed endogenous opioid and eCB activity during moderately intense exercise may be a neurobiological adaptation for endurance running, motivating and sustaining exercise through its analgesic and reward effects [4,5]. The release of eCBs and endogenous opioids generates feelings of pleasure, wellbeing, and self-transcendence, extreme forms of which are familiarly referred to as “runner’s high” [5,7]. The positive feelings associated with these effects are thought to be conducive to team bonding [8,9]. Moreover, eCBs and endorphins, a particular class of endogenous opioids, have been implicated in mammalian social bonding [10,11]. In humans specifically, there is evidence that social bonding can be mediated by endorphin release resulting from synchronous, exertive movement [12,13,14].

There is also increasing evidence that behavioral synchrony similarly affects social bonding and pain modulation. A number of experimental studies have shown that, relative to non-synchronous group activities, synchronous movement increases social bonding and prosocial behavior [15,16,17,18]–an evolutionarily important outcome of bonded relationships [12]. Recent studies have also found that, compared to solo and non-synchronous group exercise, synchronous group exercise leads to greater post-workout pain thresholds [8,19,20].

Recent research on pain processing and perception has helped to clarify further the relationship between synchrony, bonding, and exercise. Cues to social support, such as holding hands with romantic partners and viewing photos of these romantic partners, have been shown to reduce perceptions of experimentally induced pain, reduce activity in neural circuitry related to pain affect, and increase activity in neural regions known to respond to cues to safety and to modulate the threat response through top-down processes [21,22]. Indeed, cues to social support may have similar neurocognitive effects to placebo analgesic treatments, meaning that cues to cohesive social environments might act as a sort of *social placebo* [23,24,25]. In the context of physically challenging group exercise, we propose that behavioral synchrony can act as a cue to social support by increasing perceptions of togetherness and cohesion, thus activating these social support based analgesic mechanisms in a similar manner. This would suggest that synchronous exercise allows individuals to push harder, faster, for longer via increased pain thresholds and decreased perceptions of fatigue [16,26,27].

In two experimental studies, we conducted initial tests of two interrelated hypotheses on the reciprocal relationship between social bonding and exercise in two different sporting contexts. In Study 1, we tested the hypothesis that moderately intense, synchronous group exercise leads to increased bonding and cooperation among participants in a public goods game (PGG). In Study 2, we tested the hypothesis that cues to social cohesion, namely synchronous movement, allow already highly bonded athletes to perform better on a physically demanding anaerobic running test.

## Study 1

Study 1 manipulated both exercise intensity and the presence of synchrony in a 2-by-2 design. We predicted main and interaction effects of exercise intensity and behavioral synchrony, with moderately intense and synchronous exercise conditions leading to the highest levels of cooperation in the PGG among participants. We predicted that exercise intensity effects would operate via an endorphin and/or eCB effect that would be indirectly measurable via a pain threshold test [8] and that synchrony would be associated with increased perceptions of bondedness.

### Methods

#### Participants

Participants were recruited via advertisements on the University of Oxford student mailing list over an eight-week period; the advertisements stated that rowing experience was not necessary to take part in the study. In total, 71 students participated in the study. Three participants (from the same experimental session) were excluded from analysis due to failure to adhere to the experimental procedure, leaving a total of 68 participants (37 male, age range = 18–49 years, *M* = 23.05 years, *SD* = 4.70). Participants received £10 as remuneration. All participants provided written consent and the study was approved by the University of Oxford’s Central Research Committee.

#### Procedure and Materials

A 2-by-2 between-participants design was used to explore the effects of exercise intensity and synchrony during a 30-minute rowing trial on participants' subsequent bonding and cooperation in a public goods game. Conditions varied exercise intensity (low vs. moderate) and participant synchrony (synchronous vs. non-synchronous). In all conditions, participants rowed in groups of three on parallel ergometers spaced 0.5 meters apart. Groups were same or mixed-sex, determined by participant availability (53 of the 68 participants rowed in mixed-sex groups).

A male confederate who was aware of the hypotheses was used in the event of late cancelations (32 of the 68 participants rowed with the confederate); analyses did not include his responses. The confederate had no prior knowledge of the condition of each exercise session (i.e., until rowing began), after which there was no further interaction among co-participants. The confederate's participation could not be planned in advance, as conditions were counterbalanced by day and time (morning and afternoon); this resulted in uneven confederate participation across conditions, χ^2^(3) = 19.89, *p* < .001 (S1 Table).

After the initial briefing, participants underwent a pre-workout pain threshold test using a standard blood-pressure cuff measure [8] (S1 Appendix). Participants were then given a three-minute rowing practice session, after which they had the opportunity to ask questions before beginning the 30-minute trial.

Rowing intensity and synchronous rowing were varied using a metronome beat that participants listened to using wireless headphones. Participants were instructed to time their stroke according to the beat and to play a game involving stopping in unison three times throughout the experiment as a means of accenting the shared context of the activity across conditions (S2 Appendix). Participants’ heart rates were measured in beats per minute (BPM) using Polar H7 heart rate sensors.

The low intensity condition set the mean stroke rate at 16.5 strokes per minute (SPM; range: 15 SPM to 18 SPM, with stroke-rate varied in 5-minute blocks); the moderate intensity condition had a mean rate of 24 SPM (range: 22–26 SPM). These rates aimed to replicate the low and moderate intensity conditions used with treadmill runners by Raichlen, Foster, Seillier, Giuffrida, and Gerdeman [4]. In the synchronous rowing conditions all participants listened to the same metronome beat. By timing their strokes to the beat they rowed in synchrony. In the non-synchronous conditions participants heard metronome beats that differed (while maintaining mean SPM constant across participants; S2 Table). Immediately following the 30-minute trial, participants underwent the pain threshold test again before playing the PGG. In the PGG, each participant was given £5, and told that they could either keep it for themselves or give any amount of it to a group fund. Participants indicated their group fund contribution in private and anonymously in writing on a response slip (S3 Appendix). Response slips were color-coded to allow the experimenter to subsequently match responses to participant identification numbers. Participants were told that contributions would be multiplied by 1.5 and redistributed equally among all three participants regardless of whether or how much they personally contributed. They were given the opportunity to ask questions about the game and were assured their decisions would remain confidential.

Following this, participants completed the Subjective Exercise Experience Scale (SEES) [28], which contains three components measuring fatigue, psychological wellbeing, and psychological distress, respectively. They then answered six questions that have previously been used as indicators of social bonding [15,16,17]. The first question, using a modified version of the Inclusion of Other in Self (IOS) Scale [29], was “Please circle the picture that best describes your relationship to the other participants in your group” (S1 Fig). The remaining 5 questions were: “How much do you trust the other participants?”, “How similar are you to the other participants?”, “How much do you feel that you and the other participants were on the same team?”, “How much did you and the other participants cooperate during the experiment?”, and “How much do you like the other participants?”. Finally, in accordance with Reddish, Bulbulia, and Fischer [17], participants answered the following questions as a means of controlling for potential confounds: “How much do you know [each of] the other participants?” and “How difficult was the rowing trial?” All questions were answered on a 7-point scale going from 1 (*not at all*) to 7 (*very much so*).

### Results

#### Manipulation Checks

Mann-Whitney tests were used to investigate the exercise intensity and synchrony manipulations. Analyses revealed significant differences in average heart rate (BPM), *U* = 157.00, *p* < .001, *r* = -.63, fatigue, *U* = 346.50, *p* = .004, *r* = -.35, and perceived difficulty, *U* = 139.00, *p* < .001, *r* = -.67, between the low and moderate intensity conditions with the moderate intensity conditions eliciting higher average heart rates (*M* = 143.49 BPM, *SD* = 21.59 vs. *M* = 111.17 BPM, *SD* = 20.68), self-rated fatigue (*M* = 3.05, *SD* = 1.23 vs. *M* = 2.24, *SD* = 1.02), and perceived difficulty (*M* = 3.58, *SD* = 1.11 vs. *M* = 1.89, *SD* = 0.83). Analyses failed to reject the null hypothesis of no difference between the synchrony and non-synchrony conditions in perceived difficulty (non-synchronous condition, *M* = 2.85, *SD* = 1.33, vs. synchronous condition, *M* = 2.56, *SD* = 1.26; *U* = 510.50, *p* = .393). This, coupled with the experimenters’ observation that all but one group (excluded from analyses) followed their metronome beats as instructed, suggests that manipulations were followed with similar ease in the synchronous and non-synchronous conditions. Finally, the average degree to which participants knew each other was 1.48 on the 7-point scale (*SD* = 0.79) and did not differ across conditions (*F*(3, 64) = 0.40, *p* = .753).

#### PGG Contributions

Across all conditions, contributions to the group fund in the PGG ranged from £0 to £5, and 36.76% of the participants contributed all £5 to the group fund. Visual inspection of the data by experimental condition and Shapiro-Wilk normality tests revealed non-normality in PGG responses in all four experimental conditions (S3 Table). In all conditions, the PGG response data had a large proportion of responses at the maximum, £5. In other words, the response data was highly skewed.

As the available range can be viewed as censored from above—participants may have given more than £5 to the group fund in the PGG had they been given the option—we conducted a censored regression analysis. Censored regression models (also called Tobit models) [30] can offer unbiased estimates of regression coefficients when dealing with non-normal distributions that have large proportions of observations at either the minimum or maximum value (i.e., censored dependent variables).

We ran a censored regression model using the vector generalized linear model function in the VGAM package in R [31] to test first whether there was a significant exercise intensity × synchronous movement interaction on PGG contributions. Intensity, synchrony, the sex composition of the participants’ rowing group, and the self-reported measure of how well participants knew each other were also included in the model. The upper limit of the response variable was set to 5 to account for the censoring from above present in the PGG response data. This model (S4 Table) revealed no significant interaction between intensity and synchrony on PGG contributions.

Next, we omitted the non-significant intensity x synchrony interaction to create a more parsimonious censored regression model to assess the effects of intensity and synchrony on PGG contributions [32]. The other predictor variables included were the same as in the model described above. This model (S5 Table) revealed a significant positive effect of rowing intensity on PGG responses (*B* = 1.63, *SE* = 0.75, *Z* = 2.19, *p* = .029, 95% CI [0.17, 3.09], *r* = .27). The effects of the other variables included in the model were non-significant.

**Fig 1:**
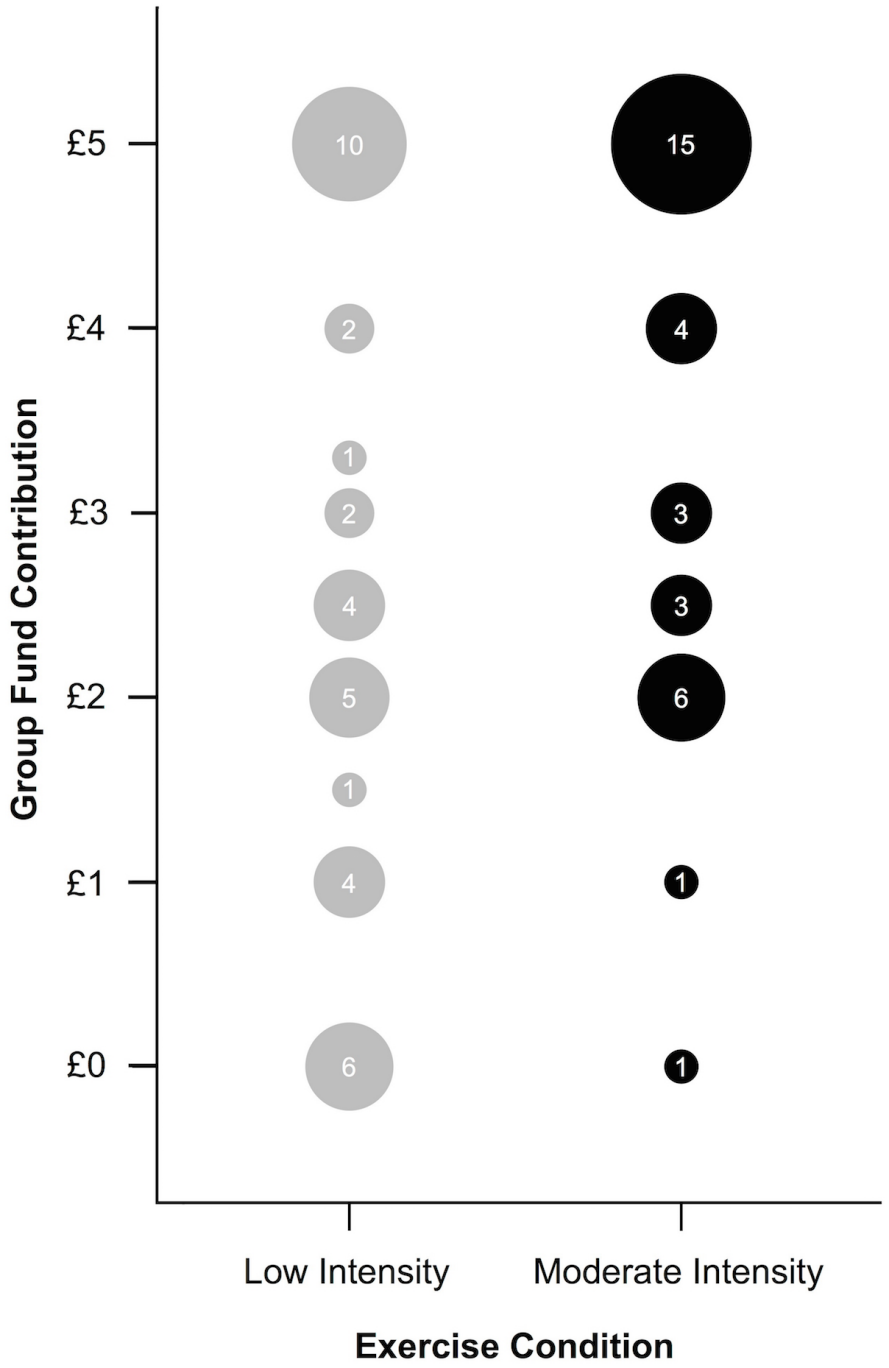
Public Goods Game Contributions by Exercise Intensity Condition. Circle area is a function of the number of participants from either the low or moderate intensity exercise conditions who contributed the given amount to the group fund in the public goods game.

The finding of a significant effect of exercise intensity on PGG contributions from the censored regression model was corroborated by analyses of the raw data (see Fig 1): the difference in mean contributions to the group fund in the PGG between the low intensity condition (*M* = £2.65, *SD* = 1.85, *n* = 35) and the moderate intensity condition (*M* = £3.65, *SD* = 1.48, *n* = 33) was £1.00 (95% CI [0.19, 1.81], Cohen’s *d* = 0.60).

#### Self-Reports and Pain Threshold Measures

Separate Mann-Whitney tests did not support previous findings on the psychological effects of exercise [5,28]: results failed to reject the tests’ null hypotheses of no difference between the exercise conditions in participants’ psychological wellbeing, *U* = 490.00, *p* = .281, psychological distress, *U* = 528.00, *p* = .528, or changes in pain thresholds, *U* = 515.50, *p* = .445. The result of a Mann-Whitney test also failed to reject the null hypothesis of no difference between synchronous and non-synchronous conditions in changes in pain threshold, *U* = 477.50, *p* = .216, again failing to replicate previous findings [8,19,20].

Based on Horn’s parallel analysis [33] and Velicer’s minimum average partial test [34], one factor, termed bondedness, was extracted from the six questions on social bonding from the post-trial questionnaire. The factor accounted for 54.38% of the variance observed in the six questions. Cronbach’s α (α = .82) indicated good internal consistency for the bondedness factor. Contrary to our predictions, a two-way analysis of covariance (S6 Table) revealed non-significant effects of exercise intensity, synchrony, and their interaction on the bondedness factor.

It also revealed a non-significant effect of how well participants knew each other, but there was a significant effect of rowing in a mixed sex group, *B* = 0.80, *SE* = 0.32, *t* = -2.48, *p* = .016, 95% CI [0.16, 1.44], *r* = .29, with mixed sex groups having higher scores on the bondedness factor than same sex groups (for a further investigation of this finding see S7 Table).

## Study 2

In Study 1, we found a significant effect of exercise intensity on PGG contributions—compared to groups of participants who rowed at a low intensity, groups of participants who rowed at a moderate intensity contributed larger amounts to the group fund in the PGG. In Study 2, we investigated the bonding-intensity link further, but in the opposite direction—do social bonds enhance exercise performance? In a within-subjects design, we used synchrony as a cue to social cohesion [16,35,36,37] and an extremely physically challenging anaerobic fitness test as the performance measure. The experiment manipulated how participants warmed up before the anaerobic fitness test: alone, non-synchronously, or synchronously with a teammate. We predicted that, relative to the solo and non-synchronous warm-up conditions, the synchronous warm-up condition would lead to the fastest performances via top-down effects of perceived social support on pain and fatigue.

### Methods

#### Participants

All members of the Oxford University Rugby Football Club (OURFC) available during a five-week testing period were recruited to participate as volunteers in the study. In total, 25 healthy male athletes agreed to participate as part of their usual training schedules (age range = 19–33 years, *M* = 23.24 years, *SD* = 2.98). Five athletes completed only two out of three conditions (three due to unavailability and two due to injury). All participants provided written consent, and the study was approved by the University of Oxford’s Central Research Committee.

#### Procedure and Materials

A within-subjects design was used to measure performance on a familiar, rugby-specific running test, the England Anaerobic Endurance Test (EAET), across three conditions. Conditions manipulated the format of the warm-up prior to the test. Participants performed warm-up exercises: (a) on their own (solo); (b) non-synchronously with a teammate; and (c) synchronously with a teammate. To control for order effects, including possible improvement in performance due to familiarity with the EAET or increased fitness over the test period, the order of the three conditions was counterbalanced across participants. Data were collected over a five-week period in accordance with athletes’ training schedules. Average rest days between participants’ first and second tests (*M* = 5.72, *SD* = 3.88) and their second and third tests (*M* = 5.10, *SD* = 4.29) did not differ significantly; *t*(19) = .181, *p* = .858 (overall average time between tests = 5.44 days, *SD* = 4.03), and participant pairs were randomly assigned for partner conditions. Participants’ maximum heart rates (BPM) during the EAET were recorded using Polar H7 heart rate sensors.

After briefing, participants underwent a pre-workout pain threshold test as in Study 1. They then completed the warm-up phase. In each warm-up manipulation participants were instructed to perform a series of repetitive full-body warm-up exercises in time to a metronome beat played through wireless headphones. The exercises were common components of a regular rugby warm-up (body weight squats, push ups, abdominal crunches, and prone alternate arm-leg extensions). In the partner conditions, participants heard either the same metronome beat (synchronous warm-up), or a different beat (non-synchronous warm-up). In the partner conditions, participants were spaced 2 meters apart from one another and oriented at a 45° angle inwards so that each player could be seen in the peripheral vision of the other player. The warm-up lasted a total of six minutes. Following the manipulation phase, a second pain threshold test was administered. Participants were then escorted to separate ends of the field to perform the EAET individually.

The EAET is designed to measure the ability to repeat bouts of high-intensity exercise with short recovery periods. It consists of five ‘sets’ of continuous running, broken up by fixed recovery times between sets, which are dictated by the time taken to complete each set (S4 Appendix). Each player’s performance in the EAET was calculated by aggregating the completion times of all five running sets.

Immediately upon completion of the EAET, a final pain threshold test was conducted. Participants then completed the Borg Scale of Perceived Exertion [38] and the SEES.

### Results

#### Manipulation Checks

We first investigated condition order, perceived exertion, and mood effects on EAET performance. A one-way ANOVA found no significant effect of condition order on performance in the EAET, *F*(2, 45.72) = 0.97,*p* = .388. One-way ANOVAs also found no significant effects of condition on the SEES (fatigue: *F*(2, 47.21) = 0.64, *p* = .530; positive wellbeing: *F*(2, 48.51) = 0.19, *p* = .830; psychological distress: *F*(2, 46.45) = 0.32, *p* = .727), rate of perceived exertion, *F*(2, 47.23) = 0.08, *p* = .921, and maximum heart rate, *F*(2, 46.12) = 0.93, *p* = .401.

#### EAET Performance

**Fig 2:**
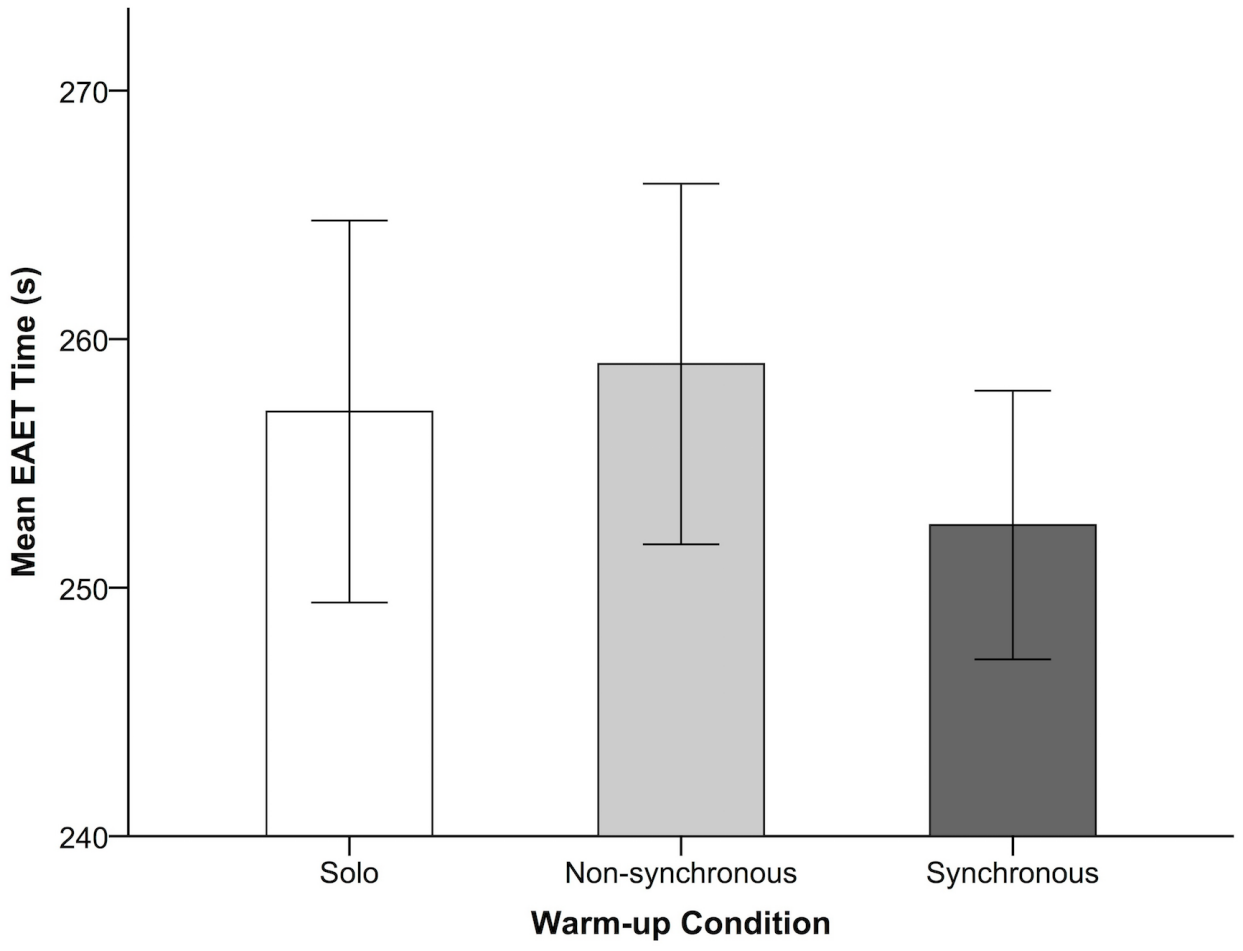
EAET Results by Warm-up Condition. Mean time (in seconds) taken to complete the EAET as a function of warm-up condition. Error bars represent 95% confidence intervals.

To test condition-wise effects on EAET performance in an unbalanced design with missing values, we employed a general linear mixed model (GLMM) fit by restricted maximum likelihood (REML) parameter estimation in SPSS Version 21 [39]. In line with our hypothesis, the GLMM revealed a significant effect of condition on performance, *F*(2, 43.68) = 4.71, *p* = .014. Bonferroni-adjusted planned pairwise comparisons with the synchronous warm-up condition as the reference category revealed a significant difference between the synchronous and non-synchronous warm-up conditions (see Fig 2). There was a significant reduction of 6.60 seconds (s) in the mean time taken to complete the test in the synchronous warm-up condition (*M* = 252.52 s, *SD* = 13.10) as compared with the non-synchronous warm-up condition (*M* = 259.12 s, *SD* = 17.06; *p* = .009, 95% CI [1.49 to 11.70], Cohen’s *d* = 0.43). The difference between the solo (*M* = 256.74 s, *SD* = 17.77) and synchronous warm-up conditions was not significant, though the trend was in the hypothesized direction (mean difference = 4.22, *p* = .115, 95% CI [-0.80 to 9.24]).

Finally, a GLMM revealed no significant effect of condition on changes in pain threshold, *F*(2, 47.46) = 0.68, *p* = .511.

## Discussion

The studies reported support our hypotheses that moderate intensity group exercise leads to cooperative social bonds among participants and that cues to group bondedness lead to a psychosocial environment where exercise ability is enhanced. Study 1 found that moderate intensity aerobic group exercise increased cooperation in a PGG as compared with low intensity group exercise. Importantly, we see our behavioral measure of the outcomes of social bonds (cooperation in the PGG) as particularly informative. Bondedness is predominantly emotional and may not be readily or accurately quantified by self-reports using analytical scales, such as those used in our questionnaire [12]. Indeed, other studies investigating cooperative social bonds have also found discrepancies between self-reports of social unity and behavioral measures of cooperation [17]. Study 2 found that behavioral synchrony, which cues social cohesion, led to a competitively meaningful increase in anaerobic ability in highly physically fit athletes. Both studies also yielded important results that we did not predict. Below we discuss the studies' findings and their limitations and offer suggestions for future research.

### Synchrony and cooperation

Accumulating experimental evidence on the link between behavioral synchrony and cooperation presents mixed findings about the conditions under which synchrony affects cooperation. Study 1 replicates findings from similar studies that found no effect of synchrony on cooperation when it was isolated from intentions to synchronize or achieve joint goals [16,17,40]. These findings are perhaps unsurprising given that most real world instances of behavioral synchrony involve explicit shared intentions to act in synchrony (e.g. dance, rowing) or, more generally, with the intention of performing the activity together collaboratively rather than individually. We suggest that fine-tuning one's movements to those of a partner or team member potentially increases collaborative attention, reinforcing a sense of trust and connection. According to the “reinforcement of cooperation model” elaborated by Reddish et al. [16], synchronous movement enhances perceptions of successful cooperation only when individuals share intentions to move in synchrony, as this positive feedback fosters greater motivation for future cooperative ventures. Further research on the interactions between synchrony, performance intensity, the perceived togetherness of the activity, shared intentionality, and social bonding is required to explore these possibilities.

Study 2 offers the first experimental evidence for the hypothesis that cues to social support enhance exercise performance; participants performed the EAET faster after warming up synchronously with a teammate as compared to warming up non-synchronously with a teammate. We view the synchrony in Study 2 as serving a cueing function. Much like the photographs of romantic partners in the pain studies referenced above, synchrony served to cue already existing close social bonds among teammates. We suggest that such cues have positive psychological effects with consequences conducive to exercise performance. In this way, close social bonds potentially act as a social placebo. Research on the placebo effect in sport has shown that social context, namely athletes’ culturally derived beliefs about the efficacy of performance related treatments, influences athletic performance [41]. It is thought that these beliefs have top-down neurological effects that ultimately allow placebo treatments (i.e., treatments that are biomedically inert) to alter muscle performance and fatigue [23,26,42].

Indeed, our findings support the hypothesis that synchrony as a cue to social support among highly bonded athletes had top-down effects on perceived fatigue via mechanisms similar to those by which the social support of close others modulates the affective experience of pain [22]. It may be that our cue of social support reduced the perceived threats of the physical and psychological difficulty associated with the EAET. This may have changed athlete’s perception of the pain and discomfort associated with fatigue, which evolved to guard against harmful overexertion [26]. This allowed participants to push harder and perform better on the EAET.

It may also be the case that both synchrony and non-synchrony have effects on performance, but in opposite directions. Non-synchrony, rather than implicitly cueing bonds between dyads, may have functioned antagonistically, serving instead to reinforce the boundaries between self and other. The possibility that synchrony (and non-synchrony) may have different psychological and behavioral effects across different social conditions is an intriguing area for follow-up research.

### Endogenous analgesia, exercise intensity, and social context

Our pain threshold and psychological wellbeing measures offered no evidence for exercise-induced endogenous opioid and/or eCB activity, or any effect of this activity on social bonding. These measures also provided no evidence that endogenous analgesic and reward mechanisms increase exercise performance or cooperation. Previous research links both exercise intensity and cues to social support to endogenous analgesia and hence activation of systems involved in pain modulation and perception, including the opioid and eCB systems [4,5,8,22,43,44] (discussed further in 'Limitations' below).

There are also at least two potential alternative interpretations of our findings. First, greater PGG cooperation in the moderate intensity exercise conditions may reflect a cognitive dissonance reduction process. Participants in the moderate intensity conditions made a greater physical effort and may have (re)interpreted this effort as an indication of their positive disposition toward making sacrifices for their exercise group, which in turn translated into increased contributions to the group fund in the PGG. Indeed, there is both experimental evidence that making sacrifices for a group increases future sacrifices for the same group [45] and quasi-experimental evidence that perceiving pain during extreme rituals predicts subsequent donations to the group with which the ritual is associated [46]. Thus, hypotheses involving cognitive dissonance offer an additional, though complementary and underexplored, explanation for our findings. Second, misattribution of arousal may explain higher scores on the bondedness factor among participants in mixed sex groups, with participants attributing arousal from exercise to arousal resulting from sexual attraction to their rowing partners [47,48]. Misattribution of arousal hypotheses would predict that the effect of mixed sex groups on bonding would be contingent on exercise intensity (i.e., arousal). However, further analyses revealed a non-significant mixed-sex group × exercise intensity interaction (see S7 Table), suggesting that it may have been a correct attribution of arousal that led participants in mixed sex groups to score higher on the bondedness factor.

### Limitations

Regarding our measures of endogenous opioid and/or eCB activity, the pressure-cuff pain threshold measure used in our studies offered a simple and accessible indirect measure of analgesic response, but may be less precise than the controlled thermal stimulus typically applied in studies on endogenous analgesia. Notwithstanding the greater methodological challenges, future studies should aim to conduct more precise measures of exercise-induced eCB and endogenous opioid activity. These studies should also seek to pinpoint the so-called ‘sweet spot’ for exercise-induced endogenous opioid and eCB release for individual participants and manipulate exercise intensity between conditions accordingly.

Further, our behavioral measure of cooperative social bonds was an economic game. However, cooperation is not exclusively motivated by the affiliative emotions that characterize bonds of social cohesion and mutual support. Further, as Fischer, Callander, Reddish, and Bulbulia [49] point out, it remains unknown to what extent monetary behaviors predict cooperation in other aspects of life. Although economic games are paradigmatic in research generally on cooperation, prosociality, and social cohesion [15,16,46,50], future research in this area should consider applying more naturalistic measures of cooperative social bonds (see, for example, [51]).

Finally, our use of a confederate in Study 1 represents a potential confound, as he was not hypothesis blind and participated unequally across conditions. As discussed, this resulted from a combination of counterbalancing the experimental conditions by day and time and from our inability to plan for cancelations that required his participation. Future studies should aim to avoid this potential confound by either using a hypothesis-blind confederate and canceling/rescheduling sessions to ensure he or she participates equally across conditions or by recruiting ‘back up’ participants to be used in the case of late cancelations. Nevertheless, we would like to emphasize that the confederate’s effect on experimental outcomes was likely negligible, as the confederate was not told the experimental condition before beginning the trial and no verbal interaction took place between participants (including the confederate) either before or during the experiment.

Finally, we emphasize the need for future research to test our hypotheses cross-culturally and in different physical group activities, exercises, and sports. This will help to elucidate the generalizability of our findings and claims about the relationships between movement, coordination, intensity, and bonding across cultures and contexts.

### Conclusion

Our studies offer general support for a reciprocal relationship between social bonding and group exercise. Specifically, Study 1 found that, compared to low intensity exercise, moderate intensity exercise led to greater cooperation among participants in a public goods game—a commonly used behavioral measure of social bonding. Study 2 found a relationship in the opposite direction, with cues to social bonds (behavioral synchrony) leading participants to perform better on a test of exercise ability. Further research is required to substantiate claims that moderately intense exercise activates endogenous opioid and eCB rewards that have positive effects on individual mood, wellbeing, and group cohesion via an exercise-induced ‘social high’. Future research will also be important for understanding how and why social context influences the performance outcomes of individuals and teams. A novel hypothesis proposed here is that cues to support act as a social placebo. According to the social placebo-in-exercise account, the presence of close others, or even mere cues to their presence (e.g., photographs, team colors) or to social cohesion (e.g., behavioral synchrony), can significantly influence the cost-benefit analyses of evolved mechanisms that optimize the use of bodily resources, such as those governing exercise performance through perceptions of pain and fatigue [26,52]. In sum, our findings open up potentially fruitful avenues of research with significant health-related applications regarding the types of group exercise most conducive to bonding, the types of social cues that enhance exercise motivation and performance, and the proximate mechanisms by which these relationships are established. Such research will also provide new answers to old questions on why, across cultures and throughout history, individuals move together to bond and bond together to move [1,2,53].

## Supporting Information

S1 Appendix Pain Threshold Test Procedure. (PDF)

S2 Appendix Rowing Game Instructions. (PDF)

S3 Appendix Public Goods Game Instruction/Response Slip. This slip was printed on a half sheet of A4 paper. Participants folded the completed slip in half before returning it to the experimenter.
(PDF)

S4 Appendix EAET Instructions. (PDF)

S1 Fig Modified IOS Scale. (PDF)

S1 Table Number of Participants Rowing with Confederate by Experimental Condition. (PDF)

S2 Table Rowing Intensity Manipulation by Condition. By following the metronome beats in their headphones participants rowed at the given strokes per minute (SPM) for each five-minute time interval during the experiment.
(PDF)

S3 Table Shapiro-Wilk Tests for PGG Response Data by Condition. (PDF)

S4 Table Results of Censored Regression with Intensity × Synchrony Interaction. (PDF)

S5 Table Results of Censored Regression without Intensity × Synchrony Interaction. (PDF)

S6 Table Results of Bondedness Factor ANCOVA. (PDF)

S7 Table Results of Bondedness Factor ANCOVA—Supplement. We included the exercise intensity × mixed sex group interaction in this model to investigate potential misattribution of arousal effects on participants’ bondedness factor scores. See the “Endogenous analgesia, exercise intensity, and social context” subsection in the Discussion section for a further consideration of the results of this model.
(PDF)
