# Supplementary material for: Social Bonds and Exercise: Evidence for a Reciprocal Relationship
Source: PLoS One. 2015 Aug 28;10(8):e0136705. doi: 10.1371/journal.pone.0136705 (PMC4552681; doi:10.1371/journal.pone.0136705)
Supplement: S1 Appendix — (PDF) [file pone.0136705.s001.pdf]

S1 Appendix. Pain Threshold Test Procedure

1. Ask the participant to sit down and to place his or her non-dominant arm on his or her thigh with palm facing upwards. Apply blood pressure cuff directly on the skin of the upper, non-dominant arm.

2. Recite the following to the participant: “I will now inflate the cuff slowly, please indicate when it becomes uncomfortable by saying ‘now’.”

3. Inflate the cuff in 10 mmHG increments at a timely, consistent pace. Stop and record the pressure in mmHG when the participant says ‘now’ or the dial reads 300 mmHG.
