# Supplementary material for: Social Bonds and Exercise: Evidence for a Reciprocal Relationship
Source: PLoS One. 2015 Aug 28;10(8):e0136705. doi: 10.1371/journal.pone.0136705 (PMC4552681; doi:10.1371/journal.pone.0136705)
Supplement: S2 Appendix — (PDF) [file pone.0136705.s002.pdf]

**S2 Appendix. Rowing Game Instructions**

These instructions were read aloud to participants before the experiment commenced:

“During the rowing trial each participant will hear the ringing of a bell in his or her headphones. Upon hearing the bell ring in your headphones, you are to move to a fully extended position (i.e., the point of maximum leg extension) and pause until your metronome beat begins again. The challenge is for the other rowers to recognize that you have stopped rowing. When you see that another group member has stopped rowing, you should immediately stop rowing yourself and move to a fully extended position. You will continue to hear your metronome beat, but you are not to begin rowing again until you see that the group member who originally stopped rowing begins rowing again. We will use a video camera to record the trial in order to measure how well group members pay attention to one another, as well as how well each individual keeps tempo with his or her metronome beat.”
