# Supplementary material for: Social Bonds and Exercise: Evidence for a Reciprocal Relationship
Source: PLoS One. 2015 Aug 28;10(8):e0136705. doi: 10.1371/journal.pone.0136705 (PMC4552681; doi:10.1371/journal.pone.0136705)
Supplement: S3 Appendix — This slip was printed on a half sheet of A4 paper. Participants folded the completed slip in half before returning it to the experimenter. (PDF) [file pone.0136705.s003.pdf]

S3 Appendix. Public Goods Game Instruction/Response Slip

GREEN

Economic Game with other participants:

You and the other participants now have £5 with which to play an economic game. With your £5 you can: (1) keep all £5 to yourself; or (2) give anywhere from £0 to £5 to a group fund while keeping any remaining money for yourself. The money given to the group fund will be multiplied by 1.5 and redistributed equally to all three participants, regardless of how much (if any) they gave to the fund.

All decisions will be kept **anonymous** and all participants have received identical instructions. Below, please write down the sum of money (if any) that you wish to keep for yourself and/or give to the group fund. **Remember the colour written at the top of this slip**. Next, fold this slip of paper in half and give it to an experimenter so that your earnings from this game can be added to your compensation for the rowing trial. To keep decisions anonymous, experimenters will not know the colour you've been assigned. At the end of the experiment please collect the envelope that is labeled with your colour. This envelope will contain your earnings. Your sum will depend on your decision and the decisions of the other participants.

Amount kept for self	Amount given to group fund	Total (this must equal £5)
		**£5**
