# Supplementary material for: Social Bonds and Exercise: Evidence for a Reciprocal Relationship
Source: PLoS One. 2015 Aug 28;10(8):e0136705. doi: 10.1371/journal.pone.0136705 (PMC4552681; doi:10.1371/journal.pone.0136705)
Supplement: S4 Appendix — (PDF) [file pone.0136705.s004.pdf]

S4 Appendix. EAET Instructions

These instructions were read aloud to participants before the experiment commenced:

“The EAET consists of 10 running shuttles. One shuttle or repetition involves starting on your stomach at the 0m line, running around the 5m pole, then back-pedaling to the 0m line and returning to your stomach before running forwards to the 10m pole and then running back to the 0m line and returning to your stomach. Finally, you will run to the 20m line and back to the 0m line. This constitutes one repetition. The time it takes you to complete one repetition will be recorded and then you will be allowed to recover for the remaining time on a 45-second running clock (one repetition takes approx. 20 seconds to complete, so you will be able to recover for the remaining 25 seconds). The EAET consists of 10 of these repetitions lumped in varying clumps as follows:

1. One repetition, followed by recovery time equal to 45 seconds minus the time taken to complete one repetition

2. Two continuous repetitions, followed by a recovery period equal to 90 seconds minus the time taken to complete two repetitions.

3. Two continuous repetitions, recovery as above.

4. Four continuous repetitions, recovery time equal to 180 seconds minus the time taken to complete these four repetitions.

5. One repetition. End of test.”
