# Supplementary material for: Social Bonds and Exercise: Evidence for a Reciprocal Relationship
Source: PLoS One. 2015 Aug 28;10(8):e0136705. doi: 10.1371/journal.pone.0136705 (PMC4552681; doi:10.1371/journal.pone.0136705)

S1 Fig. Modified IOS Scale

Please circle the picture that best describes your relationship to the other participants in your group.

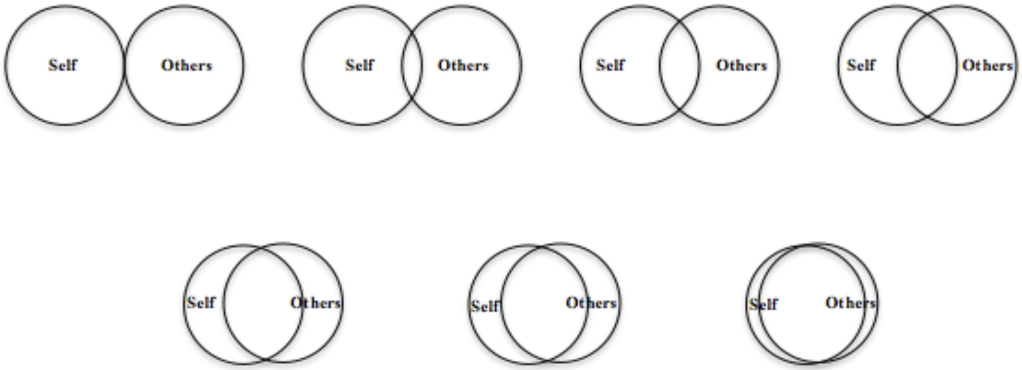

Supplement: S1 Fig — (PDF) [file pone.0136705.s005.pdf]
