# Supplementary material for: Social Bonds and Exercise: Evidence for a Reciprocal Relationship
Source: PLoS One. 2015 Aug 28;10(8):e0136705. doi: 10.1371/journal.pone.0136705 (PMC4552681; doi:10.1371/journal.pone.0136705)
Supplement: S1 Table — (PDF) [file pone.0136705.s006.pdf]

S1 Table. Number of Participants Rowing with Confederate by Experimental Condition

	Experimental Condition				
Rowed with Confederate	Low Intensity / Non-synchrony	Low Intensity / Synchrony	Moderate Intensity / Non-synchrony	Moderate Intensity / Synchrony	Total
No	12	3	6	15	36
Yes	6	14	10	2	32
