# Supplementary material for: Social Bonds and Exercise: Evidence for a Reciprocal Relationship
Source: PLoS One. 2015 Aug 28;10(8):e0136705. doi: 10.1371/journal.pone.0136705 (PMC4552681; doi:10.1371/journal.pone.0136705)
Supplement: S2 Table — By following the metronome beats in their headphones participants rowed at the given strokes per minute (SPM) for each five-minute time interval during the experiment. (PDF) [file pone.0136705.s007.pdf]

S2 Table. Rowing Intensity Manipulation by Condition

Low Intensity / Non-synchrony

	0-5 min.	5-10 min.	10-15 min.	15-20 min.	20-25 min.	25-30 min.
Participant A	18 SPM	16.5 SPM	15 SPM	18 SPM	16.5 SPM	15 SPM
Participant B	16.5 SPM	15 SPM	18 SPM	16.5 SPM	15 SPM	18 SPM
Participant C	15 SPM	18SPM	16.5 SPM	15 SPM	18 SPM	16.5 SPM

Low Intensity / Synchrony

	0-5 min.	5-10 min.	10-15 min.	15-20 min.	20-25 min.	25-30 min.
Participant A	18 SPM	16.5 SPM	15 SPM	18 SPM	16.5 SPM	15 SPM
Participant B	18 SPM	16.5 SPM	15 SPM	18 SPM	16.5 SPM	15 SPM
Participant C	18 SPM	16.5 SPM	15 SPM	18 SPM	16.5 SPM	15 SPM

Moderate Intensity / Non-synchrony

	0-5 min.	5-10 min.	10-15 min.	15-20 min.	20-25 min.	25-30 min.
Participant A	24 SPM	22 SPM	26 SPM	24 SPM	22 SPM	26 SPM
Participant B	22 SPM	26 SPM	24 SPM	22 SPM	26 SPM	24 SPM
Participant C	26 SPM	24 SPM	22 SPM	26 SPM	24 SPM	22 SPM

Moderate Intensity / Synchrony

	0-5 min.	5-10 min.	10-15 min.	15-20 min.	20-25 min.	25-30 min.
Participant A	24 SPM	22 SPM	26 SPM	24 SPM	22 SPM	26 SPM
Participant B	24 SPM	22 SPM	26 SPM	24 SPM	22 SPM	26 SPM
Participant C	24 SPM	22 SPM	26 SPM	24 SPM	22 SPM	26 SPM
