# Supplementary material for: Social Bonds and Exercise: Evidence for a Reciprocal Relationship
Source: PLoS One. 2015 Aug 28;10(8):e0136705. doi: 10.1371/journal.pone.0136705 (PMC4552681; doi:10.1371/journal.pone.0136705)
Supplement: S3 Table — (PDF) [file pone.0136705.s008.pdf]

S3 Table. Results of Shapiro-Wilk Tests on PGG Response Data by Condition

Experimental Condition	*W*	df	*p*
Low Intensity/Non-Synchrony	.880	18	.027
Low Intensity/Synchrony	.885	17	.039
Moderate Intensity/Non-Synchrony	.819	16	.004
Moderate Intensity/Synchrony	.800	17	.003
