# Supplementary material for: Social Bonds and Exercise: Evidence for a Reciprocal Relationship
Source: PLoS One. 2015 Aug 28;10(8):e0136705. doi: 10.1371/journal.pone.0136705 (PMC4552681; doi:10.1371/journal.pone.0136705)
Supplement: S4 Table — (PDF) [file pone.0136705.s009.pdf]

S4 Table. Results of Censored Regression with Intensity × Synchrony Interaction

Variable	Coeff.	*SE*	*Z*	*p*	95% CI
Intercept	3.68	1.37	2.69	.007	1.00 – 6.36
Intensity	0.98	1.13	0.87	.386	-1.24 – 3.20
Synchrony	-0.83	1.13	-0.74	.461	-3.04 – 1.38
Intensity × Synchrony	1.31	1.76	0.74	.458	-2.15 – 4.77
Mixed Sex Group	0.25	1.06	0.24	.813	-1.82 – 2.32
Prior Knowledge	-0.38	0.47	-0.82	.414	-1.31 – 0.54

Log-likelihood: $\chi^2(129) = -116.88$; Model Fit: $\chi^2(5) = 7.23$, $p = .204$; $R^2 = .03$ (McFadden's)
