# Supplementary material for: Social Bonds and Exercise: Evidence for a Reciprocal Relationship
Source: PLoS One. 2015 Aug 28;10(8):e0136705. doi: 10.1371/journal.pone.0136705 (PMC4552681; doi:10.1371/journal.pone.0136705)
Supplement: S5 Table — (PDF) [file pone.0136705.s010.pdf]

S5 Table. Results of Censored Regression without Intensity × Synchrony Interaction

Variable	Coeff.	*SE*	*Z*	*p*	95% CI
Intercept	3.11	1.12	2.78	.005	0.92 – 5.30
Intensity	1.63	0.75	2.19	.029	0.17 – 3.09
Synchrony	-0.20	0.74	-0.27	.790	-1.64 – 1.24
Mixed Sex Group	0.69	0.89	0.78	.437	-1.05 – 2.43
Prior Knowledge	-0.43	0.46	-0.95	.34	-1.33 – 0.46

Log-likelihood: $\chi^2(130) = -117.15$; Model Fit: $\chi^2(4) = 6.68$, $p = .154$; $R^2 = .03$ (McFadden's)
