# Supplementary material for: Social Bonds and Exercise: Evidence for a Reciprocal Relationship
Source: PLoS One. 2015 Aug 28;10(8):e0136705. doi: 10.1371/journal.pone.0136705 (PMC4552681; doi:10.1371/journal.pone.0136705)
Supplement: S6 Table — (PDF) [file pone.0136705.s011.pdf]

S6 Table. Results of Bondedness Factor ANCOVA

Variable	Coeff.	*SE*	*t*	*p*	95% CI
Intercept	-0.19	0.30	-0.65	.521	-0.79 – 0.40
Intensity	0.01	0.35	0.04	.969	-0.69 – 0.71
Synchrony	-0.11	0.35	-0.31	.757	-0.80 – 0.59
Intensity × Synchrony	0.08	0.53	0.15	.882	-0.98 – 1.14
Mixed Sex Group	0.80	0.32	2.48	.016	0.16 – 1.44
Prior Knowledge	0.22	0.14	1.56	.125	-1.37 – 0.50

R^2 = .18
