# Supplementary material for: Social Bonds and Exercise: Evidence for a Reciprocal Relationship
Source: PLoS One. 2015 Aug 28;10(8):e0136705. doi: 10.1371/journal.pone.0136705 (PMC4552681; doi:10.1371/journal.pone.0136705)
Supplement: S7 Table — We included the exercise intensity × mixed sex group interaction in this model to investigate potential misattribution of arousal effects on participants’ bondedness factor scores. See the “Endogenous analgesia, exercise intensity, and social context” subsection in the Discussion section for a further consideration of the results of this model. (PDF) [file pone.0136705.s012.pdf]

S7 Table. Results of Bondedness Factor ANCOVA – Supplement

Variable	Coeff.	*SE*	*t*	*p*	95% CI
Intercept	-0.16	0.29	-0.54	.592	-0.74 – 0.43
Intensity	0.23	0.27	0.86	.396	-0.30 – 0.76
Synchrony	-0.24	0.27	-0.31	.369	-0.77 – 0.29
Mixed Sex Group	0.45	0.42	1.06	.293	-0.40 – 1.29
Intensity × Mixed Sex Group	-0.74	0.64	-1.16	.252	-2.03 – 0.54
Prior Knowledge	0.19	0.14	1.32	.191	-0.10 – 0.47
R^2 = .19					
